# Towards precision medicine for brain arteriovenous malformations

**DOI:** 10.1172/JCI205086

**Published:** 2026-05-15

**Authors:** Andrew T. Hale, Adam J. Kundishora, Pazhanichamy Kalailingam, Tanyeri Barak, Phan Q. Duy, Christopher M. Ramundo, Baojian Fan, Qiang Li, Priscilla K. Brastianos, Ganesh M. Shankar, Seth L. Alper, Benjamin P. Kleinstiver, Patricia L. Musolino, Kristopher T. Kahle

**Affiliations:** 1 Department of Neurosurgery, University of Alabama at Birmingham, Birmingham, Alabama, USA.; 2 Neuroscience Institute, University of Cape Town, Cape Town, South Africa.; 3 Division of Neurosurgery, Children’s Hospital of Philadelphia, Perelman School of Medicine, University of Pennsylvania, Philadelphia, Pennsylvania, USA.; 4 Center for Genomic Medicine, Massachusetts General Hospital, Boston, Massachusetts, USA.; 5 Department of Neurosurgery, Yale University School of Medicine, New Haven, Connecticut, USA.; 6 Department of Neurosurgery, Harvard Medical School and Massachusetts General Hospital, Boston, Massachusetts, USA.; 7 Department of Medicine, Massachusetts General Hospital, Boston, Massachusetts, USA.; 8 Division of Nephrology and Vascular Biology Research Center, Beth Israel Deaconess Medical Center, Boston, Massachusetts, USA.; 9 Broad Institute of MIT and Harvard, Cambridge, Massachusetts, USA.; 10 Department of Pathology, Massachusetts General Hospital, Boston, Massachusetts, USA.; 11 Department of Pathology, Harvard Medical School, Boston, Massachusetts, USA.; 12 Department of Neurology, Harvard Medical School and Massachusetts General Hospital, Boston, Massachusetts, USA.; 13 Division of Genetics and Genomics, Boston Children’s Hospital, Boston, Massachusetts, USA.; 14 Harvard PhD Program in Neuroscience (PiN), Harvard University, Cambridge, Massachusetts, USA.

## Abstract

Recent advances in cerebrovascular genomics, single-cell biology, pharmacology, and gene editing technology are transforming our understanding of brain arteriovenous malformations (bAVMs) — a leading cause of pediatric hemorrhagic stroke. Once considered static anatomical defects, bAVMs are now recognized as dynamic, genetically driven lesions resulting from somatic mutations in *KRAS*, *BRAF*, and pathways involved in arteriovenous specification, angiogenesis, and vascular remodeling. By integrating human genetics, animal models, and endovascular innovations, researchers have uncovered convergent mechanisms that link endothelial Ras/MAPK hyperactivation to abnormal vessel growth and higher rupture risk. These insights provide a foundation for precision medicine approaches that combine molecular diagnostics — such as liquid or endoluminal biopsies — with mutation-specific pharmacotherapies and emerging CRISPR-based gene editing strategies. We suggest that genotype-guided interventions, tailored by spatial and developmental cerebrovascular context, could ultimately reclassify bAVMs from surgically incurable malformations to treatable molecular conditions.

## Introduction

Brain arteriovenous malformations (bAVMs) are direct connections between arteries and veins without an intervening capillary bed, creating high-flow, low-resistance vascular circuits (1). Estimates for sporadic bAVM incidence are approximately 0.9–1.4 per 100,000 person-years, with a prevalence of about 5–18 per 100,000 (2, 3). bAVM rupture is a leading cause of atraumatic hemorrhagic stroke in children and can result in severe morbidity, permanent neurologic disability, or death. Treatment options include microneurosurgical resection or stereotactic radiosurgery (SRS), which may involve adjunctive endovascular embolization treatments. However, many bAVMs remain untreatable due to their large size, eloquent location, and/or lack of response to SRS, among other factors. The frequent diagnosis of bAVMs in childhood and an annual rupture rate of approximately 4% (4) highlight the need for new treatment paradigms to attenuate risk of bAVM rupture. Repurposed molecularly targeted pharmacotherapeutics and/or gene editing therapies (i.e., CRISPR/Cas nucleases, prime editors, or base editors; refs. 5–7) could benefit the approximately 50%–80% of patients with sporadic *KRAS/BRAF*-mutant bAVMs (8–10) as well as patients with a bAVM-associated syndrome, where an inherited or (rarely) de novo germline mutation in the presence of a somatic “second hit” causing biallelic loss or environmental trigger (angiogenic stimuli, shear stress, transzygosity, etc.) defines AVM pathogenesis.

Here we detail the molecular mechanisms, human genetics, animal models, translational studies, and key clinical research supporting the rationale for pharmacologic and/or gene editing therapies for bAVMs. We propose a mechanistic framework for bAVM precision therapies based on our understanding of (a) signaling pathways that control angiogenesis, arteriovenous (AV) specification and zonation, and cerebrovascular (CV) cell identity; (b) spatiotemporal variations in CV single-cell developmental pathways; (c) abnormal endothelial growth, response to growth factors, and radiosensitivity; (d) animal models and human genetics related to sporadic and syndromic bAVM development; (e) common pathogenic mutations linking sporadic bAVMs and cancers with mutant-specific and pathway-modulatory therapies; and (f) tailored gene editing technologies and delivery methods that offer potential for a single, permanent correction. These insights lay the groundwork for genotype-directed precision medicine for select patients with bAVMs.

## Clinical overview of bAVMs

bAVMs present across a clinically heterogeneous spectrum with variable risk of hemorrhage, epilepsy, neurologic deficit, and systemic involvement that arise in distinct phenotypic and genetic contexts. Hereditary hemorrhagic telangiectasia (HHT1, -2, and -5) is caused by autosomal dominant inherited, or rarely de novo, mutations in *ALK1*/*ACVRL1*, *ENG*, *GDF2*, or *SMAD4* (11, 12), where somatic biallelic loss or an environmental trigger (i.e., angiogenic stimulus) defines AVM pathogenesis. HHT is typically diagnosed based on family history and identification of systemic manifestations (low-flow mucocutaneous telangiectasias, capillary malformations, and/or epistaxis) that appear in adolescence or adulthood, with bAVMs occurring in approximately 20% and 5% of HHT1 and HHT2 patients, respectively. In contrast, patients with CM-AVM — caused by somatic second-hit loss-of-function mutations in the setting of heterozygous germline mutations in *RASA1* and *EPHB4* (13) — typically present at birth or early infancy. Patients with CM-AVM have diffuse, halo-ring capillary malformations in the trunk/limbs and angioarchitecturally distinct, high-flow brain vascular malformations, including vein of Galen malformations (VOGMs), AV fistulas, or less commonly true parenchymal bAVMs. Sporadic VOGMs can present in the neonatal, infant, or early childhood periods with high-output cardiac failure, macrocephaly, and/or hydrocephalus and is associated with high morbidity and mortality. Approximately 20% of cases are caused by de novo or inherited germline variants in *EPHB4*, *RASA1*, *ACVRL1*, *NOTCH1*, *ITGB1*, and *PTPN11*, and postzygotic second-hit somatic mutations are hypothesized to define the remaining cases (14). Sturge-Weber syndrome is caused by somatic mutations in *GNAQ* and presents with facial capillary malformations (i.e., port wine stain) and seizures, secondary to hemispheric leptomeningeal vascular malformations (without formation of a true bAVM nidus), causing progressive neurologic impairment (15). In contrast, sporadic bAVMs are solitary lesions caused by somatic activating mutations in *KRAS*, and less commonly *BRAF*, that can present across the age spectrum with hemorrhage, seizures, headache, focal neurologic deficits, or incidentally where clinical manifestations are determined by variable angioarchitectural features, including size, anatomical location, and pattern of venous drainage, among others. We hypothesize that integrating knowledge of genetic drivers, molecular mechanisms, and clinical behavior in the context of angioarchitectural features across this bAVM spectrum will enable tailoring precision therapeutic strategies.

## Angiogenesis, AV specification, zonation, and CV development

CV growth, development, and remodeling occur through angiogenesis, either by sprouting from existing vessels or by partitioning into daughter branches via intussusception. These processes depend on tightly regulated molecular, genetic, and environmental cues (16). Both angiogenesis mechanisms involve proliferation, migration, patterning, cellular renewal, and morphological remodeling. Genetic and environmental modulators of angiogenesis — including shear stress (17), inflammation (18) and radiation (19) — shape CV identity, function, connections, and mechanical stability. Although multiple angiogenic phenotypes result from genetic alteration or pharmacologic manipulation of these pathways, we anticipate that specific knowledge of signaling pathways responsible for bAVM growth, maintenance, and regression will enable molecularly guided precision treatments. Aberrant spatial or temporal expression of angiogenic factors underlies bAVM histopathology (20), and risk variants in angiogenesis-associated genes may influence bAVM formation and hemorrhage (21). Therefore, framing molecularly guided bAVM treatment requires an overview of pathways regulating angiogenesis, AV specification, zonation, and CV development.

### Ephrin/Eph/RASA1.

**Figure 1:**
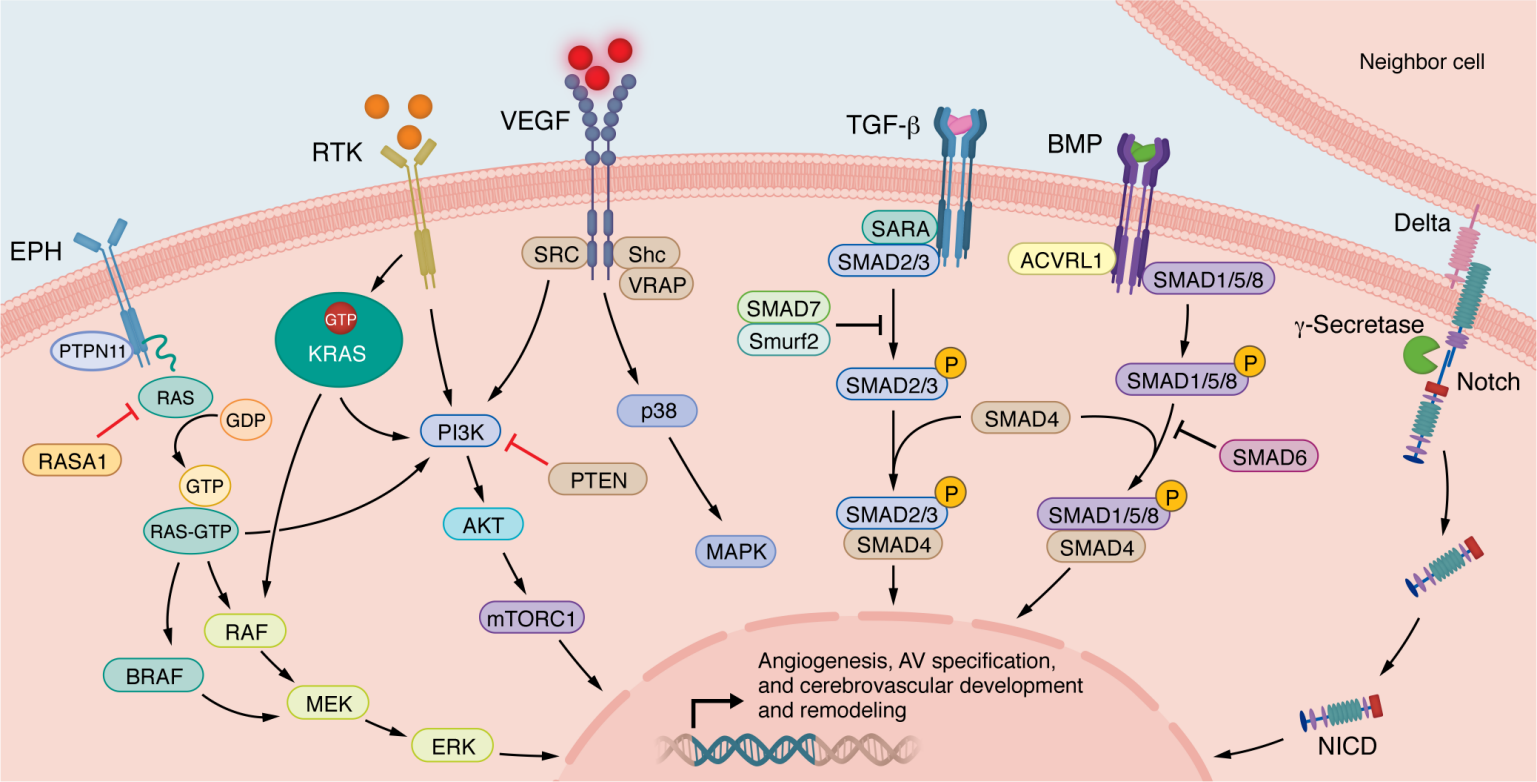
Overview of ephrin/Eph/RASA1, PI3K/AKT/mTOR, VEGF, NOTCH, TGF-β/SMAD, BRAF, and KRAS pathways. These signaling pathways underlie angiogenesis, AV specification, and CV development. Eph, ephrin; ACVRL1/ALK1, activin receptor–like kinase 1; SMAD, suppressor of mothers against decapentaplegic; SARA, SMAD anchor for receptor activation; Smurf2, SMAD ubiquitin regulatory factor 2; VRAP, VEGF receptor–associated protein; Shc, Src-like kinase C; PTEN, phosphatase and tensin homolog deleted on chromosome 10; AKT, protein kinase B; mTORC1, mammalian target of rapamycin complex 1; PTPN1, tyrosine-protein phosphatase non-receptor type I; RASA1, p120 Ras GTPase–activating protein; RAF, rapidly accelerated fibrosarcoma.

The ten EphA receptor isoforms and six EphB receptor isoforms are receptor tyrosine kinases (RTKs) that coordinate bidirectional signaling through direct interactions with the five ephrin A ligand isoforms and three ephrin B isoforms to regulate angiogenesis (Figure 1) and other cellular processes (22). Exquisite control, crosstalk, and pleiotropy among members of the ephrin/Eph pathway control endothelial cell (EC) migration and adhesion and AV differentiation, specification, and zonation in normal and pathological CV (23). Expression of ephrin B2 and EphB4 define arterial and venous ECs, respectively, during development (24). This pattern is maintained (25) but the ratio of cell types is perturbed in human bAVMs (26). Whether this perturbation is a consequence of bAVM formation or a primary mechanistic driver of bAVM pathogenesis, growth, and/or maintenance is not known. Germline SNPs in *EPHB4* have been associated with rupture risk in sporadic adult bAVMs (27), but the mechanistic basis for this observation is not clear. Our group and others have identified causative *EPHB4* loss-of-function mutations in a subset of human patients with the congenital/pediatric bAVM subtype VOGM (14, 28, 29). Heterozygous mice expressing a VOGM-specific *Ephb4* kinase domain missense variant (*Ephb4^F867L^*) displayed severe CV abnormalities only in the presence of a second variant allele or a Cre-disrupted *Ephb4* allele (14).

Downstream activation of RAS suppressor p120 Ras GTPase–activating protein (RASA1), a negative regulator of RAS signaling, is the mechanism through which EphB4 mutations regulate angiogenesis. EphB4 and RASA1 physically interact via an SH2 domain in RASA1 and a phosphorylated tyrosine residue in EphB4 (30) to limit RAS signaling in ECs (14). RAS activation also causes downstream activation of VEGF-dependent signaling (31) and is sufficient to cause bAVMs in mice (32). Somatic *RASA1* variants cause capillary malformation AVM syndrome (CM-AVM) (33, 34), characterized by high-flow vascular malformations, including systemic and intracranial AV fistulas, and, rarely, bAVM subtype VOGM (35, 36). Moreover, mice with an *Ephb4* mutation that prevents physical interaction with RASA1 but retains protein tyrosine kinase activity show normal angiogenic phenotypes (37). These data suggest, but do not prove, that EphB4 and RASA1 act within the same pathway to regulate angiogenesis. Pharmacological manipulation of RASA1 or downstream activators (e.g., mTOR) may be an appealing approach to treat genetically susceptible bAVM subtypes. However, somatic activating mutations in *EPHB4* and *RASA1* have not been identified in human patients with sporadic bAVMs.

### PI3K/AKT/mTOR.

Phosphoinositide 3-kinases (PI3Ks) are lipid kinases (class I, II, and III) that control downstream signaling pathways, including the serine-threonine kinases AKT and mTOR to control cell growth, proliferation, migration, and survival in the context of angiogenesis (Figure 1). Ligand-receptor binding or environmental stimuli such as shear stress (17) promote phospholipase C–mediated cleavage of phosphoinositide 4,5-bisphosphate (PIP_2_) to generate the second messengers diacylglycerol (DAG) and inositol-1,4,5-triphosphate (IP_3_) (38). Somatic gain-of-function mutations in *PI3KCA* have been identified in venous malformations (39) and cerebral cavernous malformations (40) in mice and humans, but not sporadic bAVMs or other bAVM subtypes. Pharmacologic inhibition of PI3K or mTOR leads to regression of PI3K-driven vascular malformations (41) and is therapeutic in mouse models of HHT (42, 43). However, in mice harboring EC-specific *Hras^G12V^* mutations, PI3K inhibition had no effect in vivo on AVM-like features (i.e., vascular dysplasia, hemorrhage, etc.) in the brain and other organs (32). Of note, to our knowledge, somatic activating mutations in *HRAS*, *NRAS*, or PI3K/AKT/mTOR pathway genes have not been identified in human patients with sporadic bAVMs. Importantly, inhibition of MEK, but not PI3K, signaling can abrogate angiogenesis through VEGF-dependent pathways in human *KRAS*-mutant bAVM ECs (10). Thus, pharmacotherapies against PI3K/AKT/mTOR are unlikely to provide therapeutic benefit for patients with sporadic bAVMs.

### VEGF.

The VEGFA-VEGFR2 interaction is the main signaling pathway driving CV angiogenesis, although other VEGF ligands (VEGFB–D) and receptors (VEGFR1–3) also have context-specific roles (Figure 1). VEGFA signaling has been extensively studied in bAVM pathogenesis in preclinical models, where overactivation of VEGFA causes CV dysmorphogenesis (44) and hemorrhage that can be attenuated by MMP9 inhibition (45). Additional support for VEGF signaling in bAVM pathogenesis is provided by mouse studies demonstrating that VEGF inhibition via treatment with the monoclonal antibody bevacizumab attenuates vascular dysplasia in mature bAVMs of *Alk1*-deficient mice (46). In contrast, two patients (genotype unknown) with sporadic bAVM treated with bevacizumab did not experience reduced bAVM volume, although the reduced VEGF serum levels observed at 26 weeks of treatment were not sustained through the end of the 52-week treatment period (47). Although no adverse systemic effects (hypertension, proteinuria, impaired wound healing, etc.) (48) of bevacizumab were reported in this case series, additional safety data are needed. In one series of patients with sporadic bAVMs, germline variants were identified in genes involved in VEGF and BMP/TGF-β pathways (49), but clear pathogenic evidence for these variants is lacking. Moreover, VEGF signaling can be augmented by shear stress (50) and iatrogenic bAVM embolization (51) through disruption of the blood-brain barrier (BBB) (52). These data highlight the highly dynamic regulation of VEGF in vivo, and suggest underlying mechanisms by which VEGF contributes to bAVM biology and treatment response. We hypothesize that VEGF signaling contributes to bAVM pathogenesis via augmentation of vascular stability and remodeling rather than as a primary genetic driver. While there is some compelling preclinical evidence, limited and variable human outcomes data, along with potential systemic toxicities, tempers enthusiasm for VEGF as a therapeutic target.

### TGF-β/SMAD.

TGF-β/SMAD signaling has both pro- and antiangiogenic roles. Clinical studies of HHT confirmed that loss-of-function variants in endoglin (*ENG*) (HHT1) or *ALK1/ACVRL1* (HHT2), both members of the TGF-β superfamily, cause the disease (53, 54). For example, ALK1/ACVRL1 is a receptor that, when bound by a ligand, phosphorylates SMAD1/5/8, enabling the latter to form a complex with SMAD4 (mutated in HHT5) and translocate to the nucleus (Figure 1). Loss of *ALK1/ACVRL1* removes the angiogenic “brake” on VEGF-mediated PI3K signaling, leading to abnormal sprouting, EC proliferation, and vascular dysplasia (55). Loss-of-function mutations in *Eng*, a transmembrane coreceptor for ALK1/ACVRL1, cause bAVMs through similar mechanisms (56–58). Mouse models lacking systemic or EC-specific *Smad4* exhibit systemic AVMs and bAVMs that respond to angiopoietin-2 inhibition (59–63). Furthermore, decreased expression of TGF-β/BMP and downstream SMAD6 has been linked to microhemorrhage in unruptured human sporadic bAVM surgical samples (64). Thus, it appears that changes in TGF-β/SMAD signaling, whether directly or via upstream regulation, may contribute to the development of bAVM pathogenesis through complex regulation of angiogenesis.

### Notch.

Notch signaling occurs through ligand-receptor interactions (DLL1/3/4, JAG1/2 binding NOTCH1–4), resulting in γ-secretase–dependent release of the Notch intracellular domain (NICD) and nuclear translocation to regulate transcription via CBF-1/RBPJ (Figure 1). Modulation of NOTCH1, -3, and -4 and RBPJ — leading to either increased or decreased Notch signaling — has been implicated in bAVM, extracranial AVM, and vascular dysgenesis in mice (65–73), but no pathogenic *NOTCH* mutations have been identified in human sporadic bAVMs. Interpretation of Notch-dependent phenotypes is complicated by downstream signaling redundancy, crosstalk, varied transcriptional programs, cell-type specificity, sequestration of Notch binding partners, differential ligand binding, and compensatory mechanisms (74). For example, deletion of the downstream Notch effector gene *Rbpj* has been shown to both cause vascular dysmorphogenesis and also rescue NOTCH4-dependent AVM formation (71–73), whereas Notch deficiency in pericytes causes extracranial AVMs and bAVMs (75). Mice lacking *EphB4* or *Efnb2* feature embryonic vascular defects similar to those observed in mice harboring *Notch1* gain-of-function mutations (67), but these phenotypes do not resemble sporadic, adult-onset bAVMs. Germline *NOTCH4* variants have also been suggested to increase risk of human bAVM formation (65), but independent replication and direct causal evidence is lacking. Additional data support shear stress– and vascular tone–mediated regulation of Notch contributing to bAVM pathogenesis (76, 77), and the therapeutic effect of radiation on bAVMs may be mediated in part by Notch signaling (78). Although Notch perturbation in mice rarely results in bAVM phenotypes independently from angiogenic modulation, these studies have been instrumental in identifying the essential role of Notch signaling in vascular development. The overlapping and convergent mechanisms with ephrin and TGF-β/SMAD suggest that these pathways may be modulated in specific bAVM biological states and in response to radiation. Thus, the role of Notch in human sporadic bAVM pathogenesis is likely dependent on genotype and environment.

### KRAS.

KRAS, a GTPase downstream of RTKs, is a major cellular regulator of growth and proliferation and has been extensively studied in oncology. The discovery of somatic mutations in *KRAS* underlying approximately 50%–80% of sporadic bAVMs (8–10) has directed attention to the role of KRAS as an angiogenesis regulator (Figure 1). KRAS activation has been shown to augment endothelial-mesenchymal transition (endoMT) through upstream modulation of TGF-β/SMAD, through a mechanism attenuated by lovastatin (79). *KRAS*-activating mutations cause downstream activation of PI3K-independent MAPK/ERK signaling (80). The prevalence of *KRAS* gain-of-function mutations in sporadic bAVMs suggests that anti-VEGF therapy may be unsuccessful, although as noted above there are very limited bevacizumab bAVM treatment data in human patients (47). *KRAS*-mutant-specific pharmacology, downstream MEK inhibition, and primary correction of pathogenic somatic mutations (or *KRAS* hyperactivation) may be viable nonsurgical treatments for human sporadic bAVMs, but additional mechanistic studies coupled with rigorous clinical trials and safety data are needed.

## Genetic basis of bAVM pathogenesis

### Human genetics of syndromic bAVMs.

Syndromes associated with bAVM development — caused by autosomal dominant inherited, or less commonly, de novo variants — include HHT1, -2, and -5 (caused by mutations in *ENG*, *ALK1/ACVRL1*, and *SMAD4*, respectively) (53, 54), CM-AVM (*RASA1* and *EPHB4* mutations) (13, 81), Parkes-Weber syndrome (*RASA1* mutations) (35), and Sturge-Weber syndrome (somatic *GNAQ* mutations) (82) (Table 1). However, most cases are incompletely penetrant and variably expressive, leading to AVMs in different locations (brain, lung, liver, etc.), where somatic second-hit loss-of-function mutations or triggers (i.e., transzygosity in modifier genes, shear stress, angiogenic stimuli, etc.; ref. 83) have been shown to determine the spatiotemporal parameters of AVM pathogenesis. Accepted mechanisms causing bAVMs in HHT include changes in PI3K activation and aberrant Notch signaling (55, 84), whereas CM-AVM cases caused by *EPHB4* and *RASA1* mutations lead to aberrant activation of RAS signaling (13, 81). However, these bAVMs differ pathologically. HHT-associated bAVMs are typically lower-flow and characterized by vessel dysplasia (more common in patients with HHT1 [~20%] than in those with HHT2 [5%]), with systemic clinical signs (i.e., mucocutaneous capillary malformations) typically appearing in adolescence or adulthood. In contrast, CM-AVM–related brain and other AVMs are high-flow and congenital or infantile, including VOGM, AV fistulas, or rarely parenchymal bAVMs that are pathologically distinct from sporadic bAVMs. Therefore, precision medicine approaches must account for the distinct genetic drivers, mechanisms, and pathophysiological characteristics of syndromic bAVMs.

### Human genetics of sporadic bAVMs.

Pathogenic somatic mutations in *KRAS*, and less frequently *BRAF*, have been found in approximately 50%–80% of surgically resected, sporadic human bAVMs (8–10). A summary of human genetic studies on sporadic bAVMs is shown in Table 2. Nikolaev et al. first identified the role of somatic activating mutations in *KRAS* (G12D, G12V, and Q61H) (10). These mutations are found at low allele frequencies (0.5%–4%), are localized to ECs, and lead to MAPK/ERK hyperactivation. Reproduction of somatic *KRAS* mutations (G12D and G12V) and the discovery of new variants (G12A, G12C, and S65_A66insDS) were reported in separate human cohorts (8, 9, 85), in which G12A and G12C were also identified in patients with spinal AVMs (9).

Germline SNPs and de novo variant (DNV) analysis of human bAVMs reveal additional possible genetic contributions, but the causative evidence for these variants is lacking. For example, germline polymorphisms in angiopoietin-like 4 (*ANGPTL4*) (86) and DNVs across 46 genes converging on endoMT and on TGF-β/SMAD and VEGF signaling pathways have been implicated in bAVM risk (49, 87). Moreover, DNV analysis of 152 sporadic bAVM proband-parent trios and 40 singletons implicated multiple genes, including *ANGPTL3* and *SLC19A3* (88), whereas an independent cohort of 60 sporadic bAVM proband-parent trios identified nonsynonymous germline variants in 46 genes enriched in vascular cell types and involved in endoMT, including *EXPH5*, *EPAS1*, and *ENG* (87). Finally, in a series of 112 additional sporadic bAVM proband-parent trios, compound heterozygous variants in 16 genes were implicated in more than one trio, including five genes (*MYLK*, *HSPG2*, *PEAK1*, *PIEZO*, and *PRUNE2*) associated with angiogenesis or vascular disease (89). However, most of these variants have not been replicated or mechanistically validated (Table 2).

## Single-cell analysis of the cerebrovasculature and bAVMs

**Figure 2:**
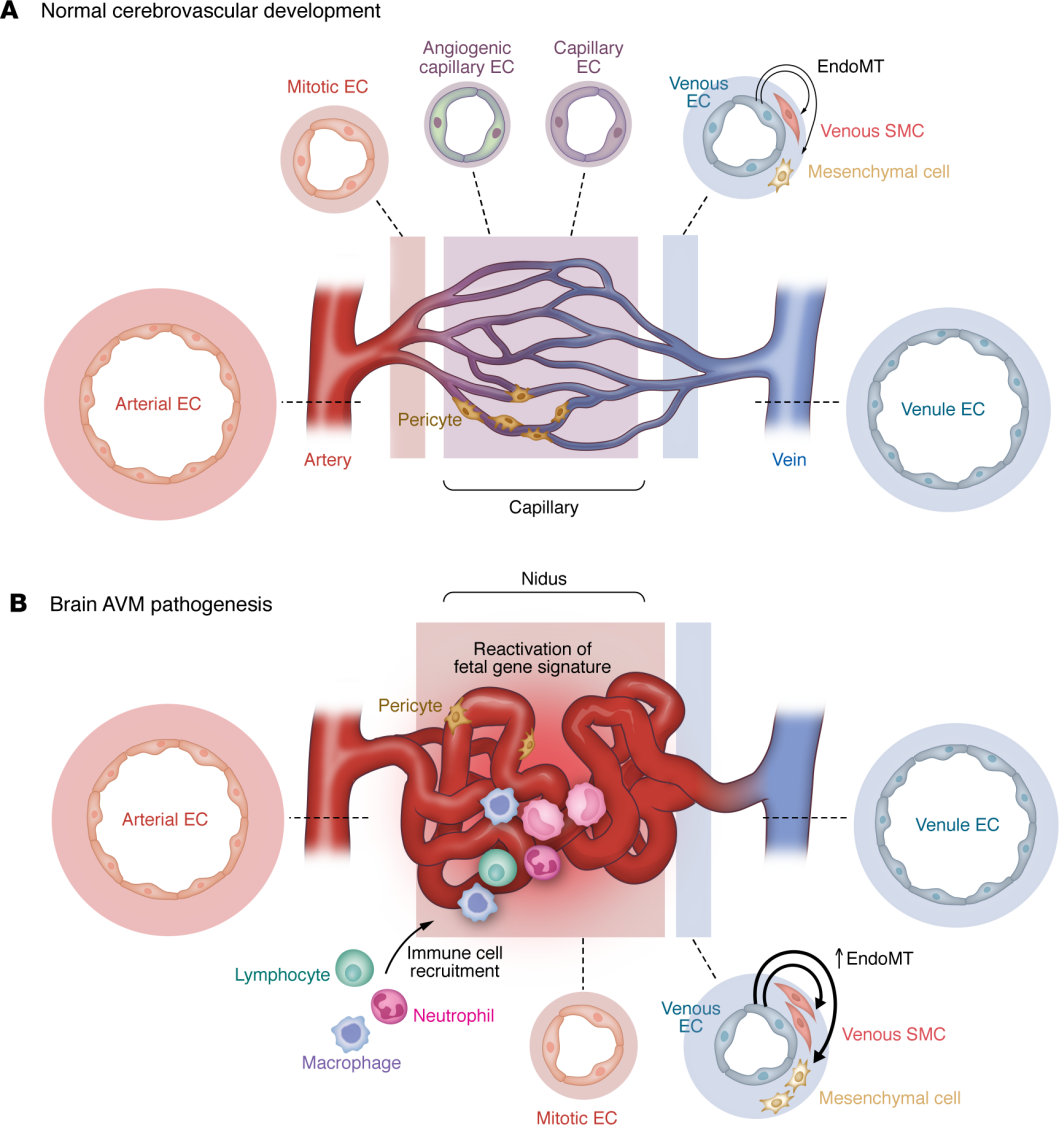
Single-cell architecture of normal CV development and bAVM pathogenesis. (**A**) Normal CV development is characterized by carefully choreographed arterial-capillary-venous development through coordinated angiogenic sprouting and endothelial specificity. Mesenchymal and vascular smooth muscle cells (SMCs) provide structural support. (**B**) Pathogenic bAVM angiogenesis is characterized by loss of the intervening capillary bed and accumulation of arterial and venous ECs. Nidal tissue is characterized by high flow, immune cell recruitment, and expression of developmentally restricted angiogenic and BBB molecular programs. EC–mural cell crosstalk, unique inflammatory cell population recruitment, and endoMT characterize bAVM rupture states.

Construction of large-scale cellular and transcriptomic atlases highlights the diversity, complexity, and heterogeneity of normal human CV system across development and disease (90–93). However, critical limitations to single-cell and spatial approaches in human bAVMs include surgical sampling bias to nidal tissue without parenchyma, reliable detection of low-frequency mutant ECs, lack of hemodynamic context, and a cross-sectional analysis without temporal developmental insights. Nonetheless, recent single-cell RNA sequencing (scRNA-seq) and supporting molecular validation experiments have identified changes within the CV in bAVMs, including accumulation of arterially fated and venous cluster ECs, presumably due to loss of capillary EC populations (a hallmark histopathological feature of bAVMs) (92). Mechanistically, Wälchli et al. demonstrated reactivation of a fetal gene signature, including multiple angiogenic pathways and angiogenic capillary markers *PVLAP* (and *ESM1*) that may be reflective of PVLAP’s role as an immature and non–BBB-competent EC marker (94). Moreover, these authors demonstrate disruption of the BBB and brain EC identities underscoring bAVM pathogenesis (92). Additional data in *Kras^G12D^*-driven bAVMs in mice also suggests that an increase in the tip cell population and angiogenic markers may be driven by androgen- and Notch-dependent signaling, providing rationale for investigation of therapeutic modulation of these pathways (95). An independent single-cell analysis of 106,853 cells across five resected bAVM specimens revealed 11 unique cell types, an increase in angiogenic potential and endoMT within the nidus, a unique immune cell signature including recruitment of myeloid cells after bAVM rupture, and modulation of hemorrhage risk via immune-mediated crosstalk with mural cells (93). Independent scRNA-seq in 17 resected bAVMs identified three unique EC clusters and one mural/fibroblast cluster (87). Finally, differential CpG methylation in *EPHB1* and *KRAS* (among other genes) has been observed in human bAVM ECs (96). Taken together, these single-cell data frame bAVMs as lesions driven by EC identity, augmented AV developmental trajectories, BBB disruption, and pathologic inflammation coordinating EC–mural cell crosstalk contributing to rupture pathogenesis (Figure 2). Future efforts to resolve the mechanistic consequences of mosaic patterning of mutant cells and integration of bAVM genotype-phenotype molecular programs will inform therapeutic strategies.

## Models to elucidate bAVM pathogenesis and treatment paradigms

Many genetically engineered mouse and zebrafish models have been developed to elucidate syndromic (Table 3) and sporadic (Table 4) bAVM pathogenesis, each offering complementary strengths and limitations (97, 98). Syndromic (i.e., HHT and CM-AVM) models have been valuable in defining mechanisms underlying human disease genes, including TGF-β/BMP changes, and the interaction between inherited genetic factors and angiogenic stimuli. Consistent with the human disease, many syndromic models display systemic AVMs prone to rupture, confounding mortality and therapeutic success specific to bAVMs. In contrast, the zebrafish models depend on transient morpholinos and exhibit embryonic development of AVM-like lesions in embryonic vessel correlates that do not fully recapitulate the postnatal human disease (Table 3).

Since most human bAVMs are sporadic and driven by somatic *KRAS* — or less commonly *BRAF* — mutations, models expressing EC-specific human variants have provided critical mechanistic and therapeutic insights (Table 4). While no current model (adeno-associated virus–mediated [AAV-mediated] delivery or Cre-inducible transgenics) replicates the mosaicism and low pathogenic allele frequency seen in human sporadic bAVMs, they have been valuable in identifying a downstream dependency on MEK hyperactivation and for testing pharmacologic and gene editing strategies. However, these models likely overestimate disease penetrance, growth kinetics, and therapeutic response owing to the challenge of targeting rare cell populations, presence of approximately 1–3 bAVMs per mouse, and CV dysplasia beyond the bAVM in many cases. Nonetheless, EC-specific expression of *Kras^G12D^* or *Kras^G12V^* in mice results in multifocal extracranial and bAVMs (80, 99), and mice harboring brain-EC-specific expression of the driver mutation have been developed to overcome this limitation (100). Treatment of *Kras^G12D^* and *Kras^G12V^* mutant mice (Cre- or AAV-driven) with trametinib (MEK inhibitor) halted bAVM progression, reduced bAVM rupture, and normalized CV architecture (100–103). In addition, mice harboring EC-specific *Kras^G12C^* expression developed systemic and bAVMs that were ameliorated with sotorasib, a chemotherapeutic targeted therapy against the *Kras^G12C^* mutation (85). Overall, both syndromic and sporadic bAVM models provide a robust and reproducible means to define bAVM disease mechanisms and assess proof-of-principle therapeutic strategies.

## Molecularly targeted therapies for bAVMs

Initial treatment approaches for bAVMs relied on antiangiogenic approaches (46, 104). However, with the discovery that somatic *KRAS* mutations cause most sporadic bAVMs, the potential of targeting KRAS activity has received special attention (105). Because *KRAS* driver mutations arise in ECs, therapeutic delivery — either small molecule or gene editing cargo — must be optimized for brain endothelial targeting. Notably, direct targeting of brain ECs does not require BBB penetration. The spatial resolution of mutant ECs to the arterial or venous side of the bAVM remains unresolved, but endovascular-enabled liquid biopsy of extracranial AVMs suggests that the draining vein may harbor the pathogenic variant (106). We hypothesize that endovascular-enabled therapeutic delivery (via transarterial or transvenous routes) may mitigate systemic pharmacologic toxicity challenges, and conjugation with liquid embolics could provide a long-term therapeutic depot. Development and/or modification of existing compounds to optimize mutation specificity, bAVM EC uptake, and long-term tolerability aimed at reprogramming bAVMs to a quiescent state (rather than complete volumetric obliteration) should be considered.

Given recent FDA recommendations on expediting testing and approval of cell- and gene-based therapies (107), precision medicine approaches for select, genetically defined bAVMs may be feasible. Nonetheless, selection of patient candidates for testing targeted therapy against bAVMs requires a multidisciplinary adjudication panel and detailed clinical monitoring. We believe that bAVM candidates for molecularly targeted therapy are those that are nonoperative (large or eloquent), actively growing, and/or radiation unresponsive. Importantly, pharmacotherapies targeting *KRAS^G12C^* (sotorasib) and *BRAF^V600E^* (vemurafenib) are currently in clinical use for cancer and approved by the FDA. Sotorasib has produced marked, and in some cases, near-complete regression of *Kras^G12C^* bAVMs in mouse models and clinically meaningful regression of facial AVMs in humans (85), while vemurafenib has been shown to obliterate AVM-like lesions in zebrafish harboring *BRAF^V600E^* mutations (108). Additional targeted agents against *KRAS^G12C^* (e.g., adagrasib, which has been shown to cross the BBB; ref. 109) and *KRAS^G12D^* are available (110, 111), but require optimization for bAVM treatment. Trametinib has been used to treat one patient with a *KRAS*-mutant spinal AVM (112) and three pediatric bAVM patients (genotype unknown) with lesions deemed too large, in eloquent location, or untreatable by radiation (113). In each case, either growth or development of high-risk features prompted the experimental treatment (113). Two of three patients demonstrated stable disease while on therapy, while one patient’s bAVM hemorrhaged during trametinib treatment, prompting an increase in dose (1 mg/day from 0.5 mg/day) (113). However, the unknown lesional mutation status and short follow-up duration limits our understanding of how treatment might have changed the natural history of these bAVMs. Importantly, analysis of the response to therapy requires consensus on what defines “success” (e.g., cessation of bAVM growth, reversal of high-risk angioarchitecture, molecular response defined by reduction in variant allele fraction by liquid biopsy, etc.). Collectively, these data suggest that *KRAS/BRAF* mutation–specific or pathway-modulatory (i.e., MEK) pharmacotherapies are promising strategies for bAVM treatment.

## Gene editing approaches for bAVMs

**Figure 3:**
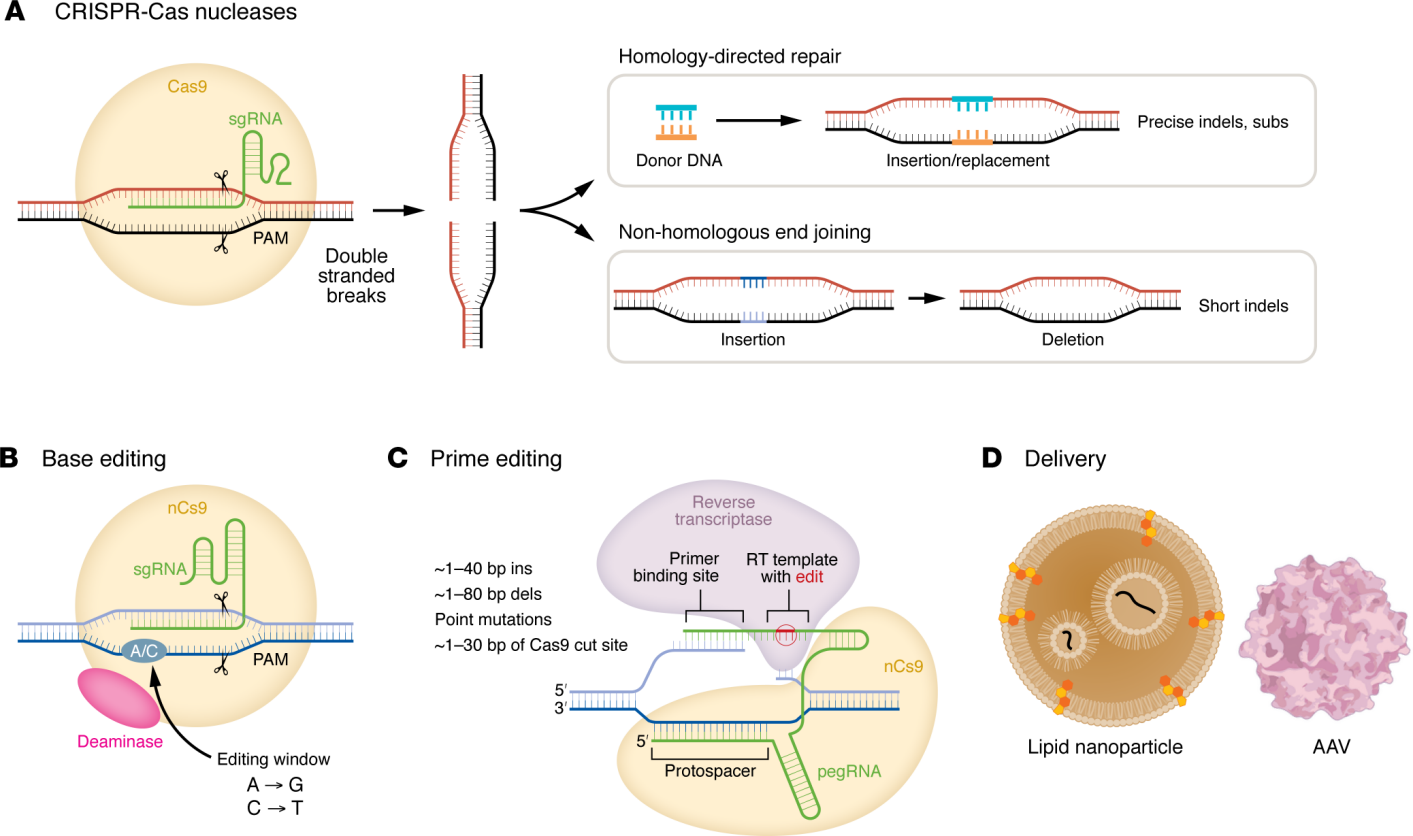
Gene editing strategies for bAVMs. (**A**) Strategies include CRISPR/Cas-based methods for gene knockout with nucleases to create insertions or deletions (indels) or templated edits via homology-directed repair. (**B** and **C**) Alternatively, more precise edits can be made with base editors and prime editors. (**D**) Delivery methods encompass lipid nanoparticles and (cerebro)vascular-specific AAVs, among others.

While molecularly targeted pharmacotherapies for bAVMs are appealing, gene editing technologies could, in principle, offer even greater precision, lasting effects, and a one-time genetic treatment (Figure 3). Limitations of CRISPR gene editing include editing specificity, targeting scope (determined by protospacer-adjacent motif [PAM] requirements), controlling and improving editing outcomes, and cell-targeted delivery methods (6, 7). Approaches to overcome specificity challenges include development of high-fidelity nuclease variants (114–116), modulating the duration of expression of the editor (117), and use of modified gRNAs (118, 119). The canonical 5′-NGG-3′ PAM sequence requirement of the Cas9 protein reduces targeting scope, but can be overcome using modified Cas9 or alternative Cas proteins with varying PAM requirements/specificities (120–130). Beyond traditional nuclease-based editing approaches, base editors can also introduce single nucleotide changes (131–134), and polymerization-based technologies, including prime editors (135) or DNA-dependent DNA polymerase–based editors (136, 137), can direct genomic DNA changes using RNA or DNA templates, respectively (Figure 3). These methods allow for more precise control of editing, particularly in the case of base or prime editing for single-point-mutation correction. Finally, additional gene editing tools include RNA editors (138) and epigenome editors (139, 140) that could be deployed in tandem with point-mutant-correction therapies (i.e., reduction of KRAS-dependent signaling) or as second-line therapies to modulate signaling pathways (i.e., VEGF, Notch, etc.) driving human bAVM maintenance, regression, response to radiation, and/or recurrence.

There are many potential challenges to gene editing for bAVMs. Specifically, human sporadic bAVMs display EC-restricted low variant allele fraction and mosaicism, requiring highly efficient and spatially restricted EC delivery. Recent advances in AAV engineering have expanded tropism to ECs in the cerebrovasculature (141–145), including a modified AAV-PR variant that transduces ECs and perivascular cells in murine models (144, 146). Lipid nanoparticles are an alternative approach, but important limitations include BBB permeability, where optimization of particle size, electrostatic properties, and selection of brain-EC-specific targeting ligands are potential clinical barriers (147–149). Nonetheless, in proof-of-concept experiments, CRISPR/Cas-mediated repression of KRAS expression has been shown to suppress bAVM growth and improve survival in *Kras^G12D^* mutant mice (99). Delivery method, immunogenicity, unintended nucleotide base conversions, introduction of genomic structural variations, and bystander edits stemming from nonspecific Cas9 binding or deaminase activity leading to unintended cellular perturbations remain key safety concerns for human clinical implementation. These data suggest that bAVMs may be suitable lesions for gene editing therapies, but a careful understanding of the disease biology, angioarchitectural markers of treatment success, and reliable detection and monitoring of the mutant EC fraction in situ will be necessary to guide the optimal gene editing approach, patient selection, and timing of treatment, among other factors.

## In situ somatic mutation detection in CV pathology

The first and critical step towards implementing molecularly targeted therapies for bAVM treatment is detecting somatic driver mutations in situ. Endoluminal biopsy is one method to obtain CV lesional tissue (150, 151). In this approach, a microcatheter delivers a coil to the target vessel wall; after a brief dwell time, the coil is retrieved and adherent cells are isolated via flow cytometry (152, 153), typically focused on isolating ECs (154–156). Endoluminal biopsy has enabled capture of lesional tissue from an intracranial fusiform aneurysm to identify a somatic driver mutation in *PDGFRB* (157). Importantly, no complications from endoluminal biopsy have been reported (154–156). Endovascular-enabled liquid biopsy, on the other hand, captures cell-free DNA from peri-AVM or intranidal blood (106), which can be seamlessly integrated with existing endovascular clinical workflows. Important limitations that need further study include safety assessments, molecular sensitivity for detecting low-frequency mutations, and technical variability in somatic DNA yield. Developing a molecular diagnostic test that targets a few “actionable” mutations could facilitate genotype-guided treatment candidacy and molecular monitoring. Given the risks associated with bAVM rupture — and the understanding of the biological mechanisms involved — the potential risks of liquid and endoluminal biopsies may be justified in carefully selected patients with high-risk, otherwise untreatable lesions, although rigorous prospective clinical studies are necessary.

## Integrated clinical-genomic framework for targeted therapy in bAVMs

**Figure 4:**
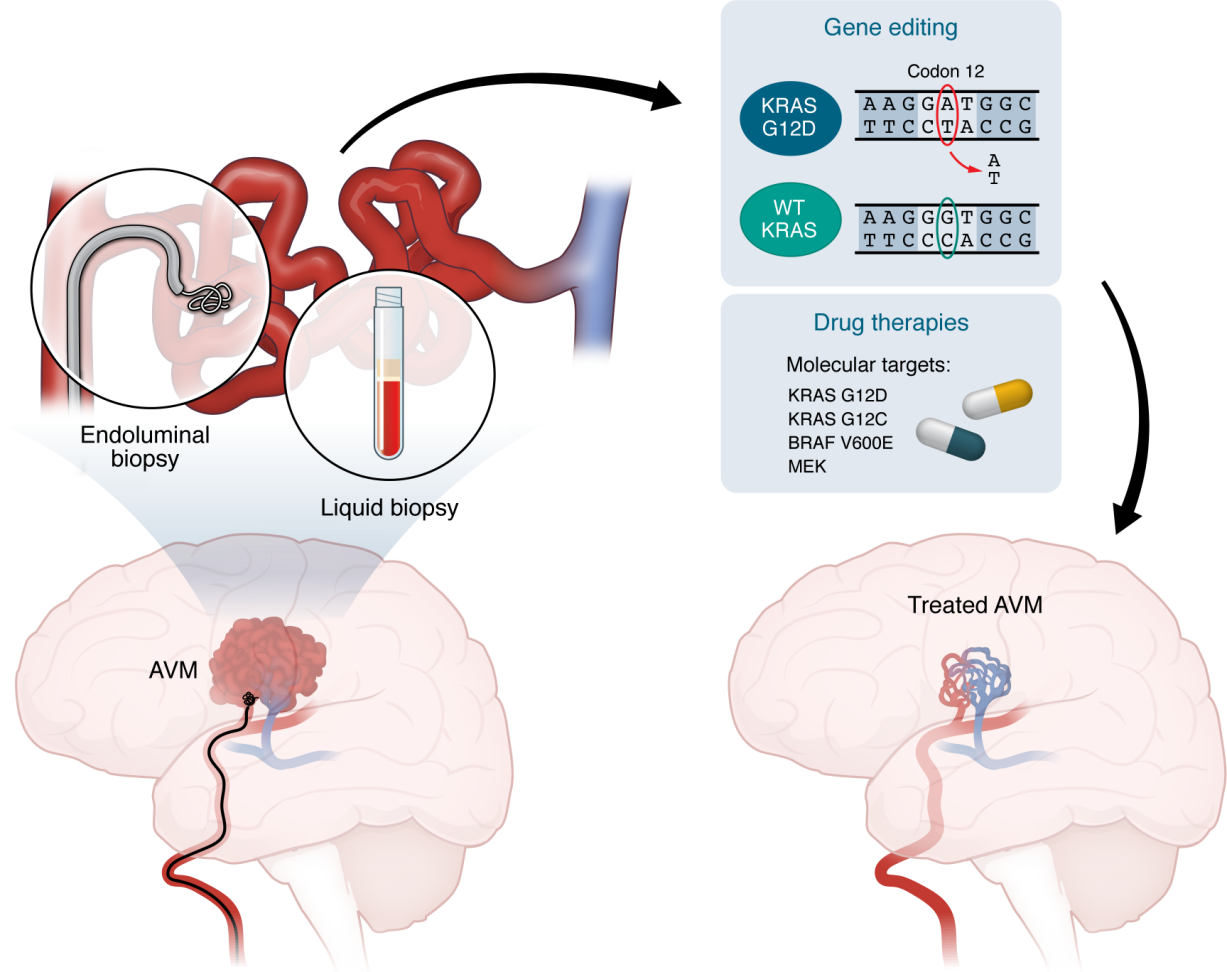
Integrated clinical-genomic framework for precision medicine in bAVMs. Molecular diagnostics enabled by endoluminal and/or liquid biopsy for in situ detection of pathogenic somatic driver mutations to guide selection of molecularly targeted pharmacotherapy (sotorasib, vemurafenib, trametinib, etc.) or to identify pathogenic alleles for gene correction therapy in vivo (i.e., bespoke base editing approaches). Endoluminal and/or liquid biopsy can then be leveraged for postoperative bAVM surveillance in tandem with neuroimaging to monitor bAVM regression over time.

How would precision medicine approaches be implemented in human patients? Rational selection of targeted therapies for bAVMs (deemed inoperable, untreatable with SRS, or persistent despite SRS) requires a minimally invasive method (i.e., liquid and/or endoluminal biopsy) for detecting somatic pathogenic variants. We suggest a precision medicine approach for bAVMs that includes (a) patient-parent trio whole-exome sequencing to detect germline mutations, (b) in situ bAVM somatic genotyping, and to guide (c) eligibility for mutation-specific or pathway modulatory agents or (e) bespoke gene editing strategies to correct pathogenic somatic mutations (Figure 4). However, consensus is needed on defining clinical, angiographic, and possibly molecular endpoints, patient selection criteria, and comparisons with natural history studies.

## Conclusions

Rapid advances in human genetics, functional genomics, and CV biology have transformed our understanding of both sporadic and inherited bAVMs. Somatic activating mutations in *KRAS* — and, less frequently, *BRAF* — are now identified as common drivers in up to 80% of sporadic, surgically resected cases, while somatic second-hit mutations in *ENG*, *ACVRL1*, *RASA1*, and *EPHB4* characterize inherited vascular syndromes variably linked to bAVMs. Alongside single-cell and spatial transcriptomic atlases and proof-of-principle animal studies, these findings create a mechanistic framework to consider precision therapy in human patients with bAVMs.

However, biological and translational gaps still exist. The timing, cadence, and hierarchy of bAVM development, genetic and environmental factors influencing growth and rupture, and the causal interaction among involved pathways are not fully understood. Many pathways implicated in model organisms often lack direct validation or relevance in human tissue, and phenotypic variability between lesions makes predicting therapeutic response more difficult. Early evidence for MEK- and KRAS-targeted treatments or CRISPR-based solutions is promising but still in the preclinical stage.

Emerging endovascular-enabled molecular diagnostics — liquid or endoluminal biopsy — may enable in situ detection of pathogenic mutations, although safety, reproducibility, and sensitivity need to be validated prospectively. Since current genomic analyses are almost entirely biased towards resected lesions, combining liquid or endovascular biopsy–enabled genotyping with angiographic anatomy could help prioritize subtypes for targeted intervention. Efforts towards clinical equipoise and multi-subspeciality coordination will be required for effective and safe translational efforts.

Ultimately, creation of an integrated genomic-phenomic taxonomy of bAVMs will redefine these malformations as treatable genetic diseases and guide genotype-directed therapeutic trials. Achieving this vision will require rigorous preclinical validation, cautious translation, and sustained collaboration across neurosurgery, interventional neuroradiology, vascular neurology, oncology, functional genomics, and bioengineering. The promise of precision medicine for bAVMs is real, but its implementation will demand both innovation and restraint.

## Conflict of interest

ATH and KTK are co-inventors on a provisional patent application, 63/841,395, detailing molecular diagnostic approaches to enable precision medicine for bAVMs. Unrelated to this work, PKB has consulted for ElevateBio, Genentech, Angiochem, Tesaro, Axiom Healthcare Strategies, InCephalo Therapeutics, Medscape, MPM Capital Advisors, Dantari Pharmaceuticals, SK Life Sciences, Pfizer, CraniUS, Kazia, Sintetica, Voyager Therapeutics, Advise Connect Inspire, and Atavistik; served on the advisory board of CraniUS and Kazia; and has received research support (institution) from Merck, Mirati, Eli Lilly, and Kinnate. BPK is a consultant for Novartis Venture Fund, Generation Bio, and Jumble Therapeutics, and is on the scientific advisory boards of Life Edit Therapeutics and Prime Medicine. BPK has a financial interest in Prime Medicine, Inc., a company developing therapeutic CRISPR/Cas technologies for gene editing. All authors’ interests were reviewed and are managed by their respective institutions in accordance with their conflict-of-interest policies.

## Funding support

This work is the result of NIH funding, in whole or in part, and is subject to the NIH Public Access Policy. Through acceptance of this federal funding, the NIH has been given a right to make the work publicly available in PubMed Central.

- NIH grants 1R01CA294793 and 5R01CA227156 (to PKB).
- NIH grants DP2CA281401 and P01HL142494 (to BPK).
- NIH grant R01NS125353 (to BPK and PLM).
- NIH grants R01NS111029, R01NS109358, and R01NS117609 (to KTK).
- National Institute on Aging grant F32AG089892 (to PQD).
- Aneurysm and AVM Foundation.
- Bee Foundation.
- Joe Niekro Foundation.
- American Association of Neurological Surgeons/Congress of Neurological Surgeons (AANS/CNS) Robert J. Dempsey, MD Research Award.
- Neurosurgery Research and Education Foundation (NREF).
- American Academy of Neurological Surgery (AAcNS).
- Society of Neurological Surgeons Neurosurgeon Scientist Training Program (to ATH).
- Breast Cancer Research Foundation (to PKB).
- National Brain Tumor Society (to PKB).
- Melanoma Research Alliance (to PKB).
- Demetra Fund from the Hellenic Women’s Club (to PKB).
- Terry and Jean de Gunzburg MGH Research Scholar Fund (to PKB).
- Kayden-Lambert MGH Research Scholar Award 2023-2028 (to BPK).

## Figures and Tables

**Table 1:**
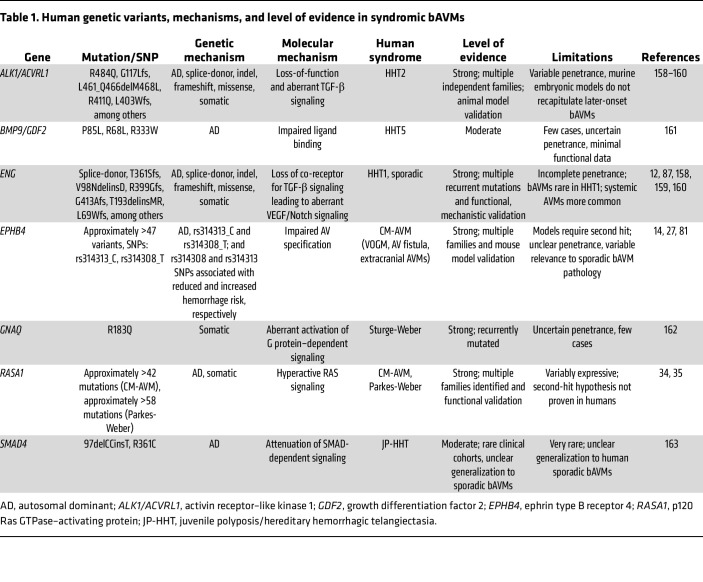
Human genetic variants, mechanisms, and level of evidence in syndromic bAVMs

**Table 2:**
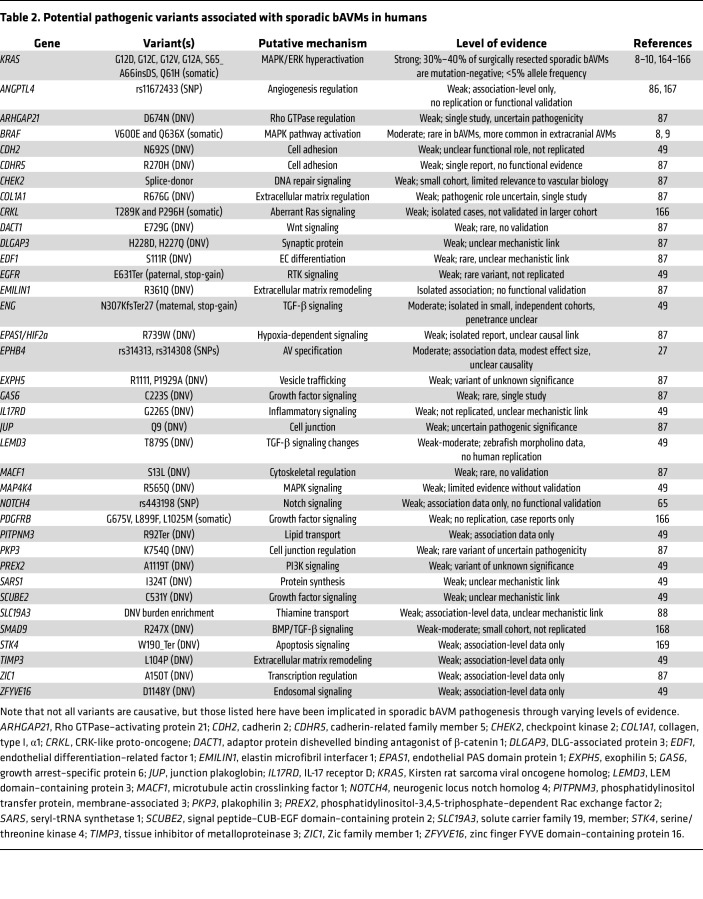
Potential pathogenic variants associated with sporadic bAVMs in humans

**Table 3:**
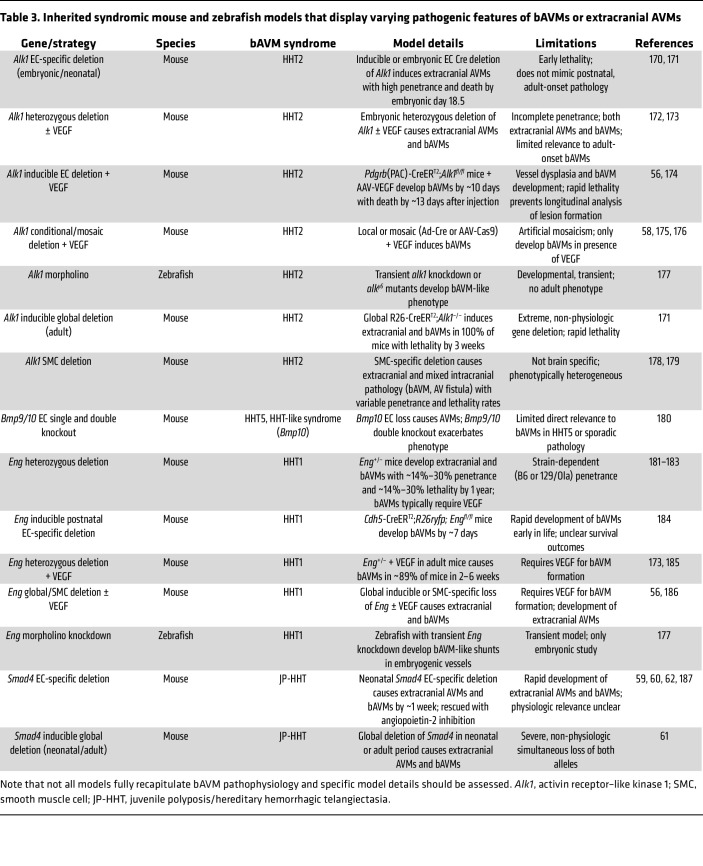
Inherited syndromic mouse and zebrafish models that display varying pathogenic features of bAVMs or extracranial AVMs

**Table 4:**
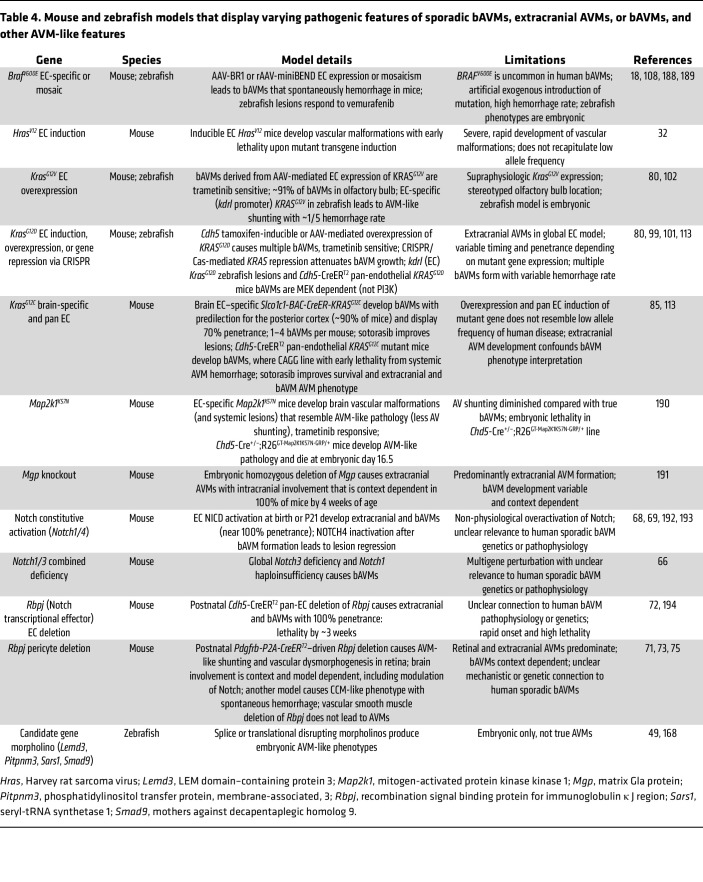
Mouse and zebrafish models that display varying pathogenic features of sporadic bAVMs, extracranial AVMs, or bAVMs, and other AVM-like features

## Version 1 05/15/2026

Electronic publication
